# Pharmacokinetics of Canakinumab in children younger than 2 years old with CAPS

**DOI:** 10.1186/1546-0096-13-S1-O38

**Published:** 2015-09-28

**Authors:** J Kalabus, P Brogan, M Hofer, J Kuemmerle-Deschner, B Lauwerys, A Speziale, R Laxer, H Sun, K Abrams, K Leon, G Junge

**Affiliations:** 1 Novartis Institutes for Biomedical Research, New Jersey, USA; 2 UCL Institute of Child Health, and Great Ormond Street Hospital NHS Foundation Trust, Department of Paediatric Rheumatology, London, UK; 3 Unité romande de rhumatologie pédiatrique, Hospitalier Universitaire Vaudois, Lausanne, Switzerland; 4 University Hospital Tuebingen, Tuebingen, Germany; 5 Cliniques Universitaires Saint-Luc and Université Catholique de Louvain, Brussels, Belgium; 6 Novartis Pharma AG, Basel, Switzerland; 7 University of Toronto, Staff Rheumatologist, The Hospital for Sick Children, Toronto, Ontario, Canada; 8 Novartis Pharmaceuticals Corporation, New Jersey, USA

## Background

Canakinumab (CAN) is indicated for the treatment of cryopyrin-associated periodic syndrome (CAPS) in patients ≥2 years of age [1]. However, information on the pharmacokinetics (PK) of CAN in patients <2 years of age is not available. Here, we present preliminary PK data from a phase III study in CAPS patients.

## Objectives

To assess the efficacy of CAN with respect to the treatment response in CAPS patients ≤4 years of age and to evaluate PK and pharmacodynamics (PD) profiling of CAN.

## Methods

CAN-naïve patients with confirmed CAPS aged 44 days to 4 years received open-label CAN 2 mg/kg every 8 weeks for 56 weeks. For NOMID patients, an initial dose of 4 mg/kg was administered. Patients who did not achieve complete response following CAN injection, or experienced a flare before the next planned administration, were eligible for dose up-titration with possible maintenance and step wise up-titration regimens of 4, 6, or 8 mg/kg s.c.

## Results

Seventeen patients, 6 patients <24 months old (44 days to 14 months; mean age = 7 months), were enrolled and administered body weight-based (2 mg/kg up to 12 mg/kg) doses of CAN s.c. every 8 weeks, with the exception of one patient who received doses of 4-6 mg/kg once weekly. Of the 6 patients <24 months old, 5 were dosed with 2 mg/kg at each dose while 1 NOMID patient started with 4 mg/kg and up-titrated to 8 mg/kg at last dose. Sixteen patients achieved a complete response, with 7 patients requiring dose escalation to achieve and/or maintain their responses. Mean dose-normalized CAN trough concentrations at steady-state in the patients <24 months old were similar across the 6 patients from 44 days to 15 months, while the range of exposures as represented by the dose normalized trough levels overlapped with the remaining 11 study patients >2 years old who received CAN doses ranging from 2 mg/kg up to12 mg/kg.

## Conclusions

Canakinumab is an effective treatment for patients with CAPS aged as young as 44 days old. The preliminary PK results demonstrated that dose-normalized canakinumab exposure in patients <2 years old was similar to patients >2 years supporting the utilization of weight-based dosing in the CAPS infantile population.
